# Pancreatic Metastasis from Intracranial Solitary Fibrous Tumor/Hemangiopericytoma Mimicking a Pancreatic Neuroendocrine Tumor: A Case Report and Focused Literature Review

**DOI:** 10.3390/curroncol33060323

**Published:** 2026-05-29

**Authors:** Xiang Kong, Fan Tong, Yaru Liu, Haochen Tang, Huizi Sha, Juan Du

**Affiliations:** 1 Department of Oncology, Nanjing Drum Tower Hospital Clinical College of Jiangsu University, Nanjing 210008, China; 2222413207@stmail.ujs.edu.cn; 2 The Comprehensive Cancer Center of Drum Tower Hospital, Medical School of Nanjing University & Clinical Cancer Institute of Nanjing University, Nanjing 210008, China; tongfan0715@163.com (F.T.); shahuizi@njglyy.com (H.S.); 3 Department of Oncology, Nanjing Drum Tower Hospital Clinical College of Nanjing University of Chinese Medicine, Nanjing 210008, China; 202411858@njucm.edu.cn; 4 Department of Oncology, Nanjing Drum Tower Hospital Clinical College of Nanjing Medical University, Nanjing 210008, China; thc6662024@163.com

**Keywords:** pancreatic metastasis, solitary fibrous tumor, intracranial SFT, STAT6, *NAB2–STAT6* fusion, PanNET

## Abstract

Solitary fibrous tumor/hemangiopericytoma (SFT/HPC) of the central nervous system is a rare mesenchymal neoplasm with a propensity for late recurrence and distant metastasis. Pancreatic metastasis from intracranial SFT/HPC is exceptionally uncommon and may mimic primary pancreatic neoplasms, particularly pancreatic neuroendocrine tumor (PanNET). We report a 52-year-old man with a documented history of recurrent intracranial SFT/HPC, historically diagnosed as hemangiopericytoma, who developed a hypervascular pancreatic tail lesion 11 years after the initial intracranial tumor diagnosis. Contrast-enhanced imaging and endoscopic ultrasound-guided fine-needle aspiration initially suggested a primary pancreatic neoplasm, including solid pseudopapillary neoplasm or PanNET, and a definitive preoperative diagnosis could not be established. Following laparoscopic resection, histopathological examination revealed a spindle-cell tumor with a rich vascular pattern. Immunohistochemistry documented STAT6 positivity, together with vimentin and Bcl-2 positivity, supporting the diagnosis of pancreatic metastasis from SFT/HPC. The patient later developed unresectable recurrent pancreatic disease and underwent stereotactic radiotherapy, showing radiological disease control at follow-up. This case highlights that pancreatic metastasis from intracranial SFT/HPC, although extremely rare, may occur after a prolonged latency period and mimic a hypervascular primary pancreatic neoplasm. In patients with a history of intracranial SFT/HPC, late metastatic disease should be considered, and definitive diagnosis relies on histopathological examination and targeted immunohistochemistry.

## Simple Summary

Pancreatic masses are usually primary pancreatic tumors, but in rare cases they may represent metastases from tumors diagnosed many years earlier. Solitary fibrous tumor/hemangiopericytoma is an uncommon mesenchymal tumor that can arise in the central nervous system and may recur or metastasize after a long interval. We report a patient who developed a hypervascular pancreatic tail lesion 11 years after treatment for an intracranial hemangiopericytoma/solitary fibrous tumor. The lesion mimicked a primary hypervascular pancreatic neoplasm, and the preoperative biopsy findings were not definitive. The final diagnosis was established after surgical resection through histopathological examination and immunohistochemistry, including STAT6 positivity. This case highlights the importance of reviewing a patient’s long-term tumor history when evaluating hypervascular pancreatic lesions and emphasizes that rare metastatic disease should be considered in patients with a prior history of intracranial SFT/HPC.

## 1. Introduction

Solitary fibrous tumor/hemangiopericytoma (SFT/HPC) is a rare mesenchymal tumor. In the central nervous system, tumors historically diagnosed as hemangiopericytoma are now recognized within the spectrum of solitary fibrous tumors [1,2]. The *NAB2–STAT6* fusion and STAT6 expression are important molecular and immunohistochemical features of SFT/HPC [3]. Although intracranial SFT/HPC is uncommon, it is clinically important because it may recur locally or metastasize extracranially after a prolonged latency period.

Secondary tumors of the pancreas are uncommon, accounting for approximately 2% of pancreatic malignancies [4,5]. The most frequently reported primary tumors include renal cell carcinoma, colorectal carcinoma, melanoma, breast carcinoma, lung carcinoma, and sarcomas. Pancreatic metastasis from intracranial SFT/HPC is exceptionally rare. Because metastatic SFT/HPC may present as a hypervascular pancreatic lesion on contrast-enhanced imaging, it can mimic primary pancreatic neoplasms, particularly pancreatic neuroendocrine tumor (PanNET). Limited preoperative sampling may further complicate the diagnosis when cytological and immunohistochemical findings overlap with those of primary pancreatic tumors.

Here, we report a rare case of pancreatic metastasis from intracranial SFT/HPC occurring 11 years after the initial intracranial tumor diagnosis. The pancreatic lesion was located in the pancreatic tail and radiologically mimicked a hypervascular primary pancreatic tumor. The final diagnosis was established by postoperative histopathological examination and immunohistochemistry. This case highlights the diagnostic pitfall of late pancreatic metastasis from intracranial SFT/HPC and emphasizes the importance of integrating long-term oncologic history with targeted pathological evaluation.

## 2. Case Presentation

A 52-year-old man with a documented history of intracranial hemangiopericytoma/SFT was evaluated for a pancreatic lesion in 2019. The primary intracranial lesion had initially been diagnosed and treated at an outside hospital in 2008, using the diagnostic terminology of “intracranial hemangiopericytoma” applied at that time. Because the original pathological materials from the 2008 primary lesion were not available for review, the WHO grade and STAT6 status of the original primary intracranial tumor could not be reassessed in the present study. During subsequent follow-up, the patient developed multiple intracranial recurrences, which were managed with repeated local treatments, including surgical resection, gamma knife radiosurgery, and external-beam radiotherapy.

In 2013, the patient underwent surgical resection of a recurrent intracranial lesion at our institution, and archival pathological materials from this procedure were available for review. Hematoxylin and eosin staining showed sheets of medium-sized tumor cells with numerous slit-like and staghorn-like vascular spaces, consistent with hemangiopericytoma/meningeal hemangiopericytoma (Figure 1A). According to the available pathological records, the recurrent intracranial lesion corresponded to approximately WHO grade III, with a Ki-67 labeling index of approximately 10%. Immunohistochemical staining performed in 2013 showed negativity for CK, EMA, GFAP, SMA, S100, and PR, and partial positivity for Bcl-2 and CD34/CD31, highlighting vascular structures. Additional immunohistochemical staining performed in 2019 on the archival 2013 specimen showed negativity for synaptophysin, chromogranin A, CD56, and SSTR2, with retained ATRX expression, arguing against a neuroendocrine tumor. These findings supported the patient’s long-standing history of recurrent intracranial hemangiopericytoma/SFT and provided important clinicopathological background for interpreting the subsequent pancreatic lesion as metastatic SFT/HPC.

In August 2019, the patient presented with intermittent postprandial upper abdominal discomfort. Laboratory tests showed that serum carbohydrate antigen 19-9 (CA19-9) was within the normal range at <2 U/mL. Abdominal ultrasonography detected a solid mass in the pancreatic tail, and contrast-enhanced computed tomography (CT) demonstrated a hypervascular lesion in the pancreatic tail (Figure 2A). Because of the marked enhancement pattern, a primary hypervascular pancreatic neoplasm, specifically pancreatic neuroendocrine tumor (PanNET), was initially considered.

Endoscopic ultrasound-guided fine-needle aspiration (EUS-FNA) of the pancreatic tail lesion was subsequently performed. The specimen showed scant mildly atypical epithelioid cell clusters within blood clots (Figure 1B). Immunohistochemistry showed weak synaptophysin positivity, chromogranin A negativity, vimentin positivity, cytokeratin negativity, a low Ki-67 index of approximately 1%, and membranous β-catenin staining. Based on these limited findings, the initial pathological impression favored solid pseudopapillary neoplasm, while PanNET remained in the differential diagnosis.

The patient subsequently underwent laparoscopic resection of the pancreatic mass with concomitant cholecystectomy in September 2019. Postoperative histopathological examination of the pancreatic lesion showed spindle-cell proliferation arranged in fascicular and storiform patterns, with staghorn-like vessels (Figure 1C). In view of the patient’s documented history of recurrent intracranial hemangiopericytoma/SFT, these findings were consistent with metastatic SFT/HPC involving the pancreas.

Immunohistochemical analysis of the resected pancreatic lesion documented STAT6 positivity, vimentin positivity, and Bcl-2 positivity, with a Ki-67 index of approximately 5%. Synaptophysin showed only focal positivity, whereas chromogranin A, CD56, cytokeratin, EMA, SMA, desmin, S100, and SOX10 were negative. CD34 highlighted vascular structures and was regarded as supportive but nonspecific rather than diagnostic in isolation. Taken together, the patient’s long-standing history of recurrent intracranial hemangiopericytoma/SFT, the characteristic spindle-cell morphology with staghorn-like vessels, and STAT6 positivity supported the diagnosis of pancreatic metastasis from intracranial SFT/HPC and argued against a primary pancreatic neuroendocrine tumor, epithelial neoplasm, smooth muscle tumor, or neural tumor.

The patient was followed regularly after surgery. In October 2024, he presented with episodic dizziness and blurred vision on standing. Non-contrast head CT demonstrated stable intracranial lesions in the right occipital and skull base regions with adjacent bone destruction. His symptoms improved after conservative treatment.

In November 2024, abdominal ultrasonography detected a solid hypoechoic mass in the right abdomen. Follow-up abdominal CT revealed marked enlargement of pancreatic lesions involving both the head and tail of the pancreas (Figure 2B), with the largest lesion measuring 9.0 cm in maximum diameter. Given the prior pathological confirmation of pancreatic metastatic SFT/HPC, these enlarging lesions were interpreted as recurrent metastatic disease.

Because the recurrent pancreatic disease was considered unresectable, the patient underwent stereotactic radiotherapy between December 2024 and January 2025, with prescribed doses of PGTV 50 Gy in 10 fractions and PTV 30 Gy in 10 fractions. Follow-up CT scans obtained in February and May 2025 demonstrated post-radiotherapy tumor shrinkage (Figure 2C,D), with the largest diameter decreasing from 9.0 cm to 6.0 cm. Because a formal RECIST-based assessment was not performed, the response was described as radiological disease control rather than assigned to a RECIST category. Ongoing surveillance is being continued.

## 3. Discussion

### 3.1. Rare Late Pancreatic Metastasis from Intracranial SFT/HPC

Intracranial solitary fibrous tumor/hemangiopericytoma (SFT/HPC) is an uncommon mesenchymal tumor characterized by a tendency for local recurrence and delayed extracranial metastasis [6]. Although the terminology and classification of SFT/HPC have evolved, the clinical relevance in the present case lies primarily in its long-term metastatic potential rather than its historical nomenclature.

The present case illustrates an exceptionally rare metastatic pattern. The patient developed a pancreatic metastasis 11 years after the initial diagnosis of intracranial hemangiopericytoma. Such a long latency period is consistent with the known biological behavior of intracranial SFT/HPC, in which distant metastases may occur many years after apparently successful local treatment.

Pancreatic metastasis from intracranial SFT/HPC is extremely uncommon. In the limited cases reported in the literature, pancreatic involvement usually occurred after a prolonged interval, often more than 10 years after the diagnosis of the primary intracranial tumor. Therefore, the present case reinforces the need for long-term surveillance in patients with intracranial SFT/HPC and highlights that a new pancreatic lesion in this clinical context should not automatically be regarded as a primary pancreatic neoplasm.

### 3.2. Diagnostic Pitfall: Mimicry of Hypervascular Pancreatic Neoplasms

The major diagnostic pitfall in this case was that the pancreatic metastasis radiologically and cytologically mimicked a primary pancreatic neoplasm. On contrast-enhanced CT, the lesion appeared as a hypervascular mass in the pancreatic tail, which initially raised suspicion for a pancreatic neuroendocrine tumor (PanNET). This interpretation was clinically reasonable, as PanNETs commonly present as well-circumscribed hyperenhancing pancreatic masses.

Preoperative EUS-FNA further contributed to the diagnostic uncertainty. The specimen contained only scant mildly atypical epithelioid cell clusters, and immunohistochemistry showed weak synaptophysin positivity, chromogranin A negativity, vimentin positivity, cytokeratin negativity, a low Ki-67 index, and membranous β-catenin staining. Based on these limited findings, the initial pathological impression favored solid pseudopapillary neoplasm (SPN), while PanNET remained in the differential diagnosis. However, the findings were not definitive for either entity.

This case illustrates an important limitation of imaging and limited preoperative sampling in rare metastatic pancreatic lesions. Hypervascularity alone is not specific for PanNET, and focal or weak synaptophysin positivity should be interpreted cautiously when other neuroendocrine markers, such as chromogranin A and CD56, are negative. Similarly, SPN was not fully supported because β-catenin staining was membranous rather than nuclear, and the clinical context was atypical. A concise comparison of the main diagnostic considerations in this case is summarized in Table 1.

For this reason, the differential diagnosis should be guided not only by imaging and cytology, but also by the patient’s long-term oncologic history. In a patient with a prior history of intracranial hemangiopericytoma/SFT, a new hypervascular pancreatic lesion should prompt consideration of late metastatic SFT/HPC, even after a long disease-free interval. In the present case, definitive diagnosis required postoperative histopathological examination and targeted immunohistochemistry. These diagnostic uncertainties underscore the importance of an appropriately selected immunohistochemical panel, particularly STAT6, in establishing the diagnosis of metastatic SFT/HPC.

### 3.3. Pathological Confirmation and Clinical Implications

Pathological confirmation was essential in the present case because neither imaging nor preoperative EUS-FNA provided a definitive diagnosis. The resected pancreatic lesion showed spindle-cell proliferation with a rich vascular pattern, which, in the context of the patient’s documented history of intracranial hemangiopericytoma, raised strong suspicion for metastatic SFT/HPC. Immunohistochemical analysis further supported this diagnosis by documenting STAT6 positivity, together with vimentin and Bcl-2 positivity. In contrast, markers supporting a primary pancreatic neuroendocrine tumor or epithelial neoplasm, including chromogranin A, CD56, and cytokeratin, were negative.

STAT6 is particularly important in the diagnosis of SFT/HPC because nuclear STAT6 expression is widely used as an immunohistochemical surrogate for the characteristic *NAB2–STAT6* fusion and helps distinguish SFT/HPC from histological mimics [12]. In the present case, STAT6 positivity was the key immunohistochemical finding supporting the diagnosis of metastatic SFT/HPC. By contrast, CD34 expression should be interpreted with caution. Although CD34 positivity is frequently observed in SFT/HPC, it is not specific and should not be weighted as definitive diagnostic evidence in isolation. Therefore, in this case, CD34 was regarded only as a supportive and nonspecific finding, whereas STAT6 positivity, tumor morphology, and the patient’s prior oncologic history were considered more diagnostically relevant.

Several limitations should be acknowledged. First, detailed first-hand histopathological and immunohistochemical materials from the original intracranial tumor were unavailable because the primary lesion had been diagnosed in 2008. Therefore, the STAT6 status of the primary intracranial lesion could not be assessed in the present study. Second, although STAT6 positivity of the pancreatic lesion was documented in the original pathology report, archival limitations restricted additional pathological material review and prevented retrieval of a representative STAT6-stained image of sufficient quality for publication. Therefore, an anonymized excerpt of the original pathology report is provided in Supplementary Figure S1. Third, *NAB2–STAT6* fusion gene analysis was not performed. Nevertheless, the diagnosis was supported by the patient’s documented history of intracranial hemangiopericytoma, the long latency period, the characteristic histopathological morphology of the pancreatic lesion, and the immunohistochemical profile of the pancreatic tumor.

Pancreatic metastasis from intracranial SFT/HPC is extremely rare. To contextualize the present case, we reviewed the limited published cases of pancreatic metastasis from intracranial SFT/HPC. As summarized in Table 2, the reported cases generally showed a long latency interval between the primary intracranial tumor and pancreatic metastasis, ranging from approximately one decade to more than two decades. These observations support the need for prolonged surveillance in patients with intracranial SFT/HPC, even after apparently successful local control.

The clinical implication of this case is that a newly detected hypervascular pancreatic lesion in a patient with a history of intracranial SFT/HPC should raise suspicion for late metastatic disease, even after a prolonged disease-free interval. Definitive diagnosis should rely on histopathological examination and an appropriately selected immunohistochemical panel rather than imaging findings alone. Recognition of this rare metastatic pattern may help avoid misclassification as a primary pancreatic tumor and support the need for prolonged surveillance in patients with intracranial SFT/HPC.

## 4. Conclusions

Pancreatic metastasis from intracranial SFT/HPC is exceptionally rare but should be considered in patients with a compatible oncologic history, particularly when a hypervascular pancreatic lesion is identified after a long disease-free interval. This case demonstrates that metastatic SFT/HPC may closely mimic primary pancreatic neoplasms such as PanNET on imaging and cytology. Accurate diagnosis depends on the integration of long-term clinical history, histopathological evaluation, and targeted immunohistochemistry, with STAT6 positivity serving as a key diagnostic finding. Because late recurrence and distant metastasis may occur many years after the initial diagnosis, prolonged surveillance is warranted.

## Figures and Tables

**Figure 1:**
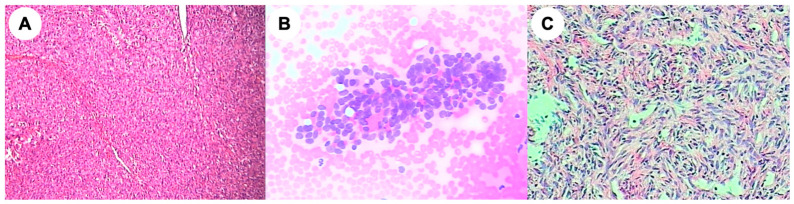
Histopathological and cytological features of the recurrent intracranial lesion and pancreatic lesion. (**A**) Histopathological features of the recurrent intracranial lesion. Hematoxylin and eosin staining of the recurrent intracranial lesion resected at our institution in 2013 showed sheets of medium-sized tumor cells with numerous slit-like and staghorn-like vascular spaces (magnification: ×200). (**B**) Preoperative EUS-FNA specimen from the pancreatic tail lesion showing scant mildly atypical epithelioid cell clusters within blood clot. The initial pathological impression favored solid pseudopapillary neoplasm, with PanNET considered in the differential diagnosis because of weak synaptophysin positivity (magnification: ×300). (**C**) Hematoxylin and eosin staining of the resected pancreatic lesion showing spindle-cell proliferation arranged in fascicular and storiform patterns, with staghorn-like vessels (magnification: ×200).

**Figure 2:**
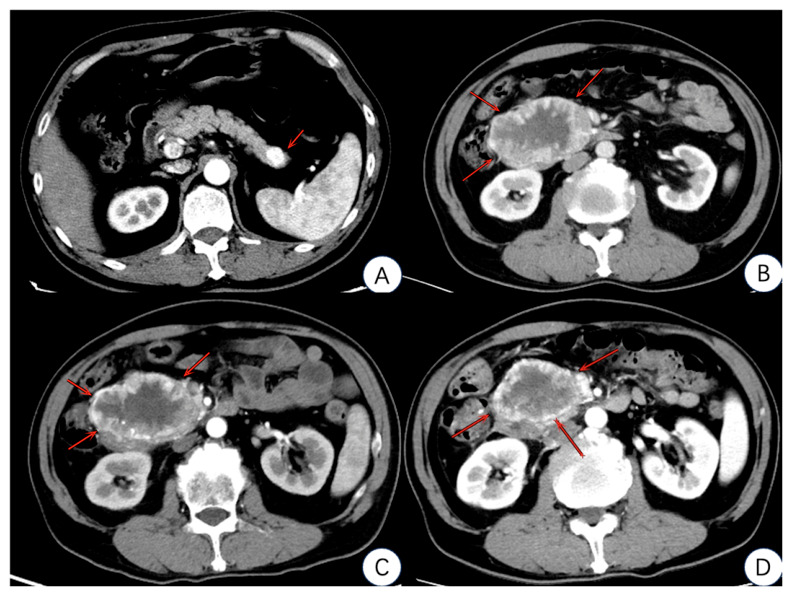
Serial CT imaging of pancreatic lesions before and after radiotherapy. (**A**) Initial abdominal CT in 2019 shows a hypervascular mass in the pancreatic tail. (**B**) Follow-up CT in 2024 reveals recurrent pancreatic lesions involving the pancreatic head and tail. (**C**) Post-radiotherapy CT in February 2025 shows decreased lesion size. (**D**) Follow-up CT in May 2025 shows sustained disease control.

**Table 1:** Key differential diagnostic considerations for the hypervascular pancreatic lesion in this case.

Feature	PanNET [7]	SPN [8]	Metastatic SFT/HPC	RCC Metastasis [9,10,11]
Imaging	Hyperenhancing solid mass	Solid-cystic or encapsulated pancreatic mass	Well-demarcated hypervascular mass	Hypervascular enhancing lesion
Clinical context	May be functional or nonfunctional pancreatic tumor	More common in young women	History of intracranial SFT/HPC, often with long latency	History of renal cell carcinoma
IHC markers	Synaptophysin+, chromogranin A+, CD56+	Nuclear β-catenin+, CD10+, vimentin+	STAT6+, variable CD34/Bcl-2+	PAX8+, CD10+, CAIX+
Relevance to present case	Considered radiologically, but not supported by CgA/CD56 negativity	Initially favored based on EUS-FNA but β-catenin was membranous rather than nuclear	Supported by prior history of intracranial SFT/HPC, morphology, and nuclear STAT6 positivity	Less likely without RCC history or supportive renal-lineage markers

Abbreviations: PanNET, pancreatic neuroendocrine tumor; SPN, solid pseudopapillary neoplasm; SFT/HPC, solitary fibrous tumor/hemangiopericytoma; RCC, renal cell carcinoma; IHC, immunohistochemistry; CgA, chromogranin A; CAIX, carbonic anhydrase IX.

**Table 2:** Reported cases of pancreatic metastasis from intracranial SFT/HPC and key clinicopathological features.

Author/Year	Age/ Sex	Primary Site	Latency, Years	Pancreatic Involvement	Treatment	Key Pathology/IHC and Outcome
Hiraide 2014 [13]	65/M	Cerebellum	24	Two pancreatic metastases: body and tail	DP	CD34+; CD99+. Alive, NED at 1 yr.
Yi 2023 [14]	68/M	Intracranial	14	Solitary head lesion	PPPD + RT	STAT6+; CD34+; EMA+. Alive, NED at 20 mo.
Yamashita 2019 [15]	58/F	Intracranial	27	Solitary tail lesion, cystic appearance	DP	Diffuse nuclear STAT6+; CD34+; CD99+; Bcl-2+. Meningeal recurrence at 4 yrs; no abdominal recurrence.
Gao 2023 [16]	64/F	Intracranial	10	Large pancreatic lesion with hemorrhage	PP + splenectomy; later TT/IO	Vim+; SMA+; cytoplasmic β-catenin+; focal CD34/CD99/S-100+; STAT6− pancreatic lesion. Alive with slowly progressing/stable systemic disease.
Present case	52/M	Intracranial	11	Tail metastasis; later recurrent head and tail lesions	LR + SRT	Nuclear STAT6+; Bcl-2+; vimentin+; CD34 highlighted vessels. Alive, RDC at 6 mo.

Abbreviations: SFT/HPC, solitary fibrous tumor/hemangiopericytoma; IHC, immunohistochemistry; M, male; F, female; yr, year; yrs, years; mo, months; DP, distal pancreatectomy; PPPD, pylorus-preserving pancreaticoduodenectomy; RT, radiotherapy; PP, partial pancreatectomy; TT, targeted therapy; IO, immunotherapy; LR, laparoscopic resection; SRT, stereotactic radiotherapy; NED, no evidence of disease; RDC, radiological disease control; EMA, epithelial membrane antigen; SMA, smooth muscle actin; Vim, vimentin.

## Data Availability

The data supporting the findings of this case report are included in the article. Further inquiries can be directed to the corresponding author.

## Supplementary Materials

The following supporting information can be downloaded at: https://www.mdpi.com/article/10.3390/curroncol33060323/s1. Supplementary Figure S1. Anonymized excerpts from the original pathology reports of the pancreatic lesion resected in 2019 and the recurrent intracranial lesion resected in 2013.

## Abbreviations

The following abbreviations are used in this manuscript:

SFT	solitary fibrous tumor
HPC	hemangiopericytoma
PanNET	pancreatic neuroendocrine tumor
CT	computed tomography
CA19-9	carbohydrate antigen 19-9
EUS-FNA	endoscopic ultrasound-guided fine-needle aspiration
RDC	radiological disease control
PGTV	planning gross tumor volume
PTV	planning target volume
SPN	solid pseudopapillary neoplasm
RCC	renal cell carcinoma
