# Supplementary figures and images for: Pancreatic Metastasis from Intracranial Solitary Fibrous Tumor/Hemangiopericytoma Mimicking a Pancreatic Neuroendocrine Tumor: A Case Report and Focused Literature Review
Source: Curr Oncol. 2026 May 29;33(6):323. doi: 10.3390/curroncol33060323 (PMC13298388; doi:10.3390/curroncol33060323)

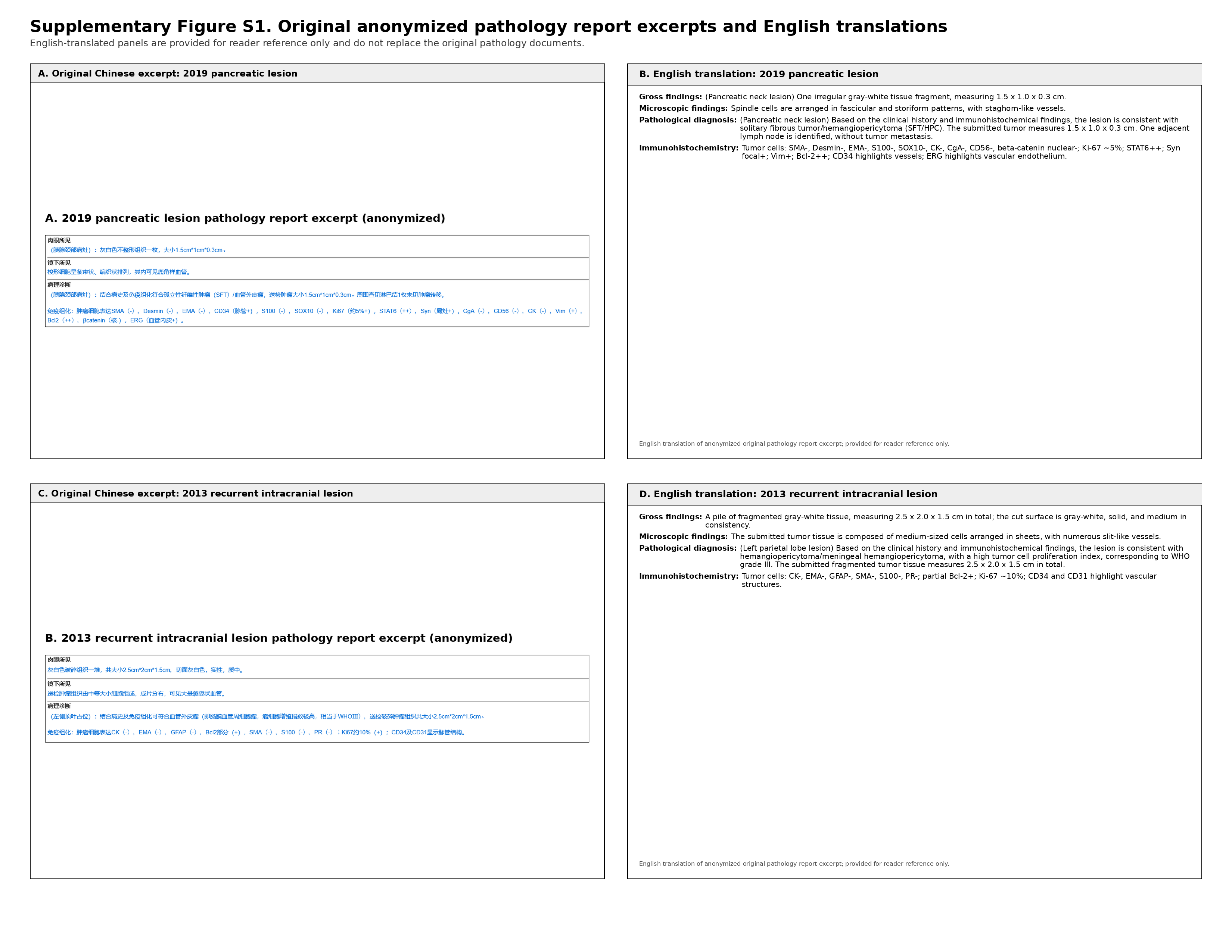

Supplement: Supplementary file 1 [file curroncol-33-00323-s001.zip › curroncol-4329963-supplementary.png]
